# Highly Resolved and Robust Dynamic X‐Ray Imaging Using Perovskite Glass‐Ceramic Scintillator with Reduced Light Scattering

**DOI:** 10.1002/advs.202003728

**Published:** 2021-06-02

**Authors:** Wenbo Ma, Tingming Jiang, Ze Yang, Hao Zhang, Yirong Su, Zeng Chen, Xinya Chen, Yaoguang Ma, Wenjuan Zhu, Xue Yu, Haiming Zhu, Jianbei Qiu, Xu Liu, Xuhui Xu, Yang (Michael) Yang

**Affiliations:** ^1^ State Key Laboratory of Modern Optical Instrumentation College of Optical Science and Engineering International Research Center for Advanced Photonics Key Laboratory of Excited State Materials of Zhejiang Province Zhejiang University Hangzhou Zhejiang 310027 China; ^2^ Faculty of Material Science and Engineering Kunming University of Science and Technology Kunming Yunnan 650000 China; ^3^ Center for Chemistry of High‐Performance and Novel Materials Department of Chemistry Zhejiang University Hangzhou Zhejiang 310027 China

**Keywords:** perovskites, quantum dots, scintillator, spatial resolution, X‐ray imaging

## Abstract

All‐inorganic perovskite quantum dots (QDs) CsPbX_3_ (X = Cl, Br, and I) have recently emerged as a new promising class of X‐ray scintillators. However, the instability of perovskite QDs and the strong optical scattering of the thick opaque QD scintillator film imped it to realize high‐quality and robust X‐ray image. Herein, the europium (Eu) doped CsPbBr_3_ QDs are in situ grown inside transparent amorphous matrix to form glass‐ceramic (GC) scintillator with glass phase serving as both matrix and encapsulation for the perovskite QD scintillators. The small amount of Eu dopant optimizes the crystallization of CsPbBr_3_ QDs and makes their distribution more uniform in the glass matrix, which can significantly reduce the light scattering and also enhance the photoluminescence emission of CsPbBr_3_ QDs. As a result, a remarkably high spatial resolution of 15.0 lp mm^−1^ is realized thanks to the reduced light scattering, which is so far a record resolution for perovskite scintillator based X‐ray imaging, and the scintillation stability is also significantly improved compared to the bare perovskite QD scintillators. Those results provide an effective platform particularly for the emerging perovskite nanocrystal scintillators to reduce light scattering and improve radiation hardness.

## Graphical abstract

Europium (Eu) doped perovskite QD glass ceramic works as perfect scintillator because of high light yield, superior transparency, and hence significantly suppressed optical crosstalk, as well as the protection of glass matrix. Eventually the record‐high imaging resolution of 15.0 lp mm^−1^ is achieved, the radiation hardness is also significantly improved.

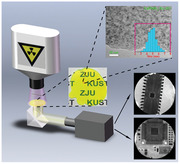

X‐ray has been widely used in probing the inside information of condensed matter subjects non‐destructively, enabling broad applications in safety inspection, medical radiography, defect inspection, etc.^[^ ^1^, ^2^, ^3^, ^4^, ^5^, ^6^, ^7^, ^8^, ^9^ ^]^ There are two approaches to detect X‐ray: 1) direct conversion of X‐ray photons into electrical signal; 2) indirect conversion by using scintillators to convert X‐ray photons to visible photons first and then be detected by photodiode. In the past few years, perovskite semiconductors have demonstrated great potentials in direct X‐ray detection due to their exceptional properties such as large X‐ray attenuation coefficient, large mobility‐lifetime product (*µτ*), and low‐cost solution process.^[^ ^10^, ^11^, ^12^, ^13^, ^14^, ^15^, ^16^, ^17^, ^18^ ^]^ Recently, all‐inorganic CsPbX_3_ (X = Cl, Br, I) perovskite scintillators have been explored due to their large X‐ray absorption efficiency, intense radioluminescence (RL), low X‐ray detection limit, and fast light decay,^[^ ^1^, ^2^, ^3^, ^4^, ^17^, ^18^, ^19^ ^]^ which make them highly competitive to the commercial scintillators, for example, CsI:TI^[^ ^20^ ^]^ and LuAG:Ce based scintillators.^[^ ^21^ ^]^ In addition, perovskite‐like metal halides featuring self‐trapped exciton emissions have revealed good X‐ray scintillation with large Stokes shift and high light yield.^[^ ^22^, ^23^ ^]^ One advantage of inorganic perovskite as scintillator is that there is no need to grow as big crystals as many conventional scintillator to obtain good scintillation yield, in contrast, the scintillation of CsPbBr_3_ nanocrystal is several orders of magnitudes stronger than CsPbBr_3_ single crystal.^[^ ^1^ ^]^ However, this unique feature also brings dark sides. First, it requires the perovskite nanocrystal film to be at least several hundred micrometers to ensure sufficient X‐ray absorption, such thick film is opaque and the light scattering severely affects the imaging resolution. Second, the stability issue could become even more severe when perovskite crystals shrink to nanometer size due to the massively increased surface/bulk ratio. In addition, the water soluble lead of perovskite nanocrystal is always a risk to human and environment.^[^ ^13^, ^24^ ^]^ Growing QDs inside inorganic glass has been proven as an effective strategy to prevent the degradation and enhance the environmental stability of II–VI and IV–VI QDs,^[^ ^25^, ^26^, ^27^, ^28^ ^]^ as well as CsPbX3 QDs showing applications in white LED,^[^ ^29^, ^30^, ^31^, ^32^, ^33^, ^34^, ^35^, ^36^ ^]^ lasering,^[^ ^36^ ^]^ and 3D laser printing.^[^ ^37^ ^]^ Here, to resolve those above concerns, we in situ grew inorganic perovskite QDs inside transparent and robust matrix as a glass‐ceramic (GC) scintillator, which is inexpensive to fabricate and can be easily scaled up. The incorporation of small amount of Eu improves the crystallinity of CsPbBr_3_ QDs and alters its size distribution, which further enhances the scintillator performance in terms of spatial resolution and light yield. Finally, our CsPbBr_3_:Eu GC scintillator with significantly reduced light scattering enables X‐ray images with high spatial resolution of 15 lp mm^−1^, even superior to commercial CsI (TI) scintillator and delivers impressive stability under continuous X‐ray illumination and thermal stressing in humid air.

The CsPbBr_3_ QDs grown in glass matrix was prepared from the transparent precursor glass (PG) by melt‐quenching method, whose XRD pattern of PG (**Figure** **1**a) showed diffuse hump without any diffraction peaks due to its amorphous character. Subsequently the PG was annealed at 500 °C and the CsPbBr_3_ QDs were precipitated inside it, as evidenced by the XRD peaks (Figure 1a) that are well indexed to pure CsPbBr_3_ cubic phase (JCPDS No. 54–0752). TEM bright filed image of CsPbBr_3_ GC (Figure 1b) presents dark spherical CsPbBr_3_ nanocrystals embedded in glass with average diameter of ≈11 nm. And high‐resolution TEM image (Figure 1c) shows clear lattice fringe with interplanar distance between two adjacent crystal planes of ≈0.348 nm, in agreement with the (110) plane of cubic CsPbBr_3_ crystal. Interestingly, the nanocrystals shrink to the typical size of quantum dots (≈5 nm) with the incorporation of Eu, and the size distribution is more uniform shown in Figure 1d. We reason that the doped Eu^3+^ likely acts as a nucleation agent which can promote rapid growth and precipitation of CsPbBr_3_, thus decrease the crystal size and improve the uniformity. In order to conform this, we monitored the in situ growth of CsPbBr_3_ QDs induced by high‐energy electron beam inside PG as shown in Figures S1 and S2, Supporting Information. The TEM images demonstrate the CsPbBr_3_ QDs are crystallized more rapidly, easily, and uniformly inside Eu doped PG. Since the thickness of the GC is about 2 mm, the perovskite loading among glass is determined to be ≈116 µm based on molar ratio of source materials and their corresponding density, which is similar to the reference nanocrystal film of ≈100 µm thickness. Since Rayleigh scattering intensity is proportional to *d* ^6^ (diameter of the dots), the light scattering for the Eu doped CsPbBr_3_ QDs GC, with optimized doping ratio of 1.5% (Figure 1e) can be significantly eliminated, which ultimately translates to reduced signal crosstalk in photodiode array and therefore improve the image resolution. Figure 1f presents the photographs of CsPbBr_3_ GC with and without Eu^3+^ dopant as well as conventional CsPbBr_3_ nanocrystal film, demonstrating distinctly improved transparency for CsPbBr_3_:Eu GC visually, mainly because of the reduced scattering rather than the difference of absorption. We applied Eu doping strategy to grow other perovskite QDs (CsPbCl_3_ and CsPbI_3_) in the same glass matrix and found the sizes of perovskite QDs also uniformly distributed in the matrix as shown in Figure S1, Supporting Information, which demonstrates the effectiveness of this approach.

**Figure 1:**
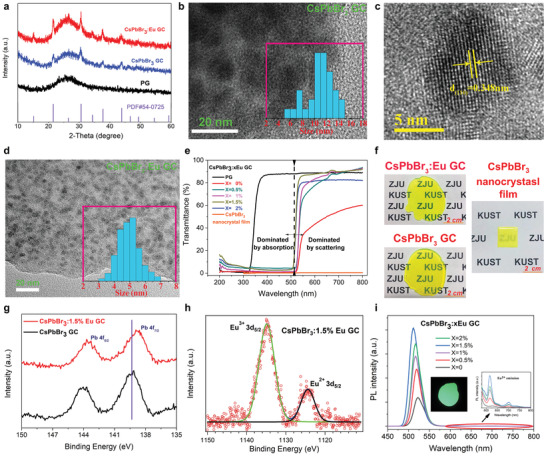
Fabrication and characterization of CsPbBr_3_ quantum dots (QDs) inside transparent amorphous matrix. a) XRD patterns of precursor glass (PG), CsPbBr_3_ QDs with and without Eu dopant in GC. b) TEM image of CsPbBr_3_ QDs without Eu dopant in glass (quantum dot size distribution shown in inset). c) HRTEM image of CsPbBr_3_ GC. d) TEM image of CsPbBr_3_:Eu GC (quantum dot size distribution shown in inset). e) Transmittance spectra of CsPbBr_3_:*x*Eu QDs in glass as a function of Eu concentration (transmittance spectrum of PG measured as a reference). f) Photographs of CsPbBr_3_ GC, CsPbBr_3_:1.5%Eu GC (optimal concentration of Eu dopant) and conventional CsPbBr_3_ nanocrystal film with thickness of about 100 µm (CsPbBr_3_ nanocrystals and polystyrene are uniformly mixed with the ratio of 1:5). g) Pb 4f XPS spectra of CsPbBr_3_ GC with and without Eu dopant. h) Eu 3d_5/2_ XPS spectrum of CsPbBr_3_:1.5%Eu GC. i) Photoluminescence (PL) spectra of CsPbBr_3_:*x*Eu GC as a function of Eu concentration under 365 nm UV excitation (photograph of CsPbBr_3_:1.5%Eu GC emission and enlargement of Eu^3+^ emission shown in the inset). PL spectra is measured with integrating sphere to ensure fair comparison

X‐ray photoelectron spectroscopy (XPS) measurements were carried out to study the doping effect of Eu^3+^ ions. The binding energy of Pb 4f_7/2_ shifts from 139.2 eV for CsPbBr_3_ GC to 138.73 eV for CsPbBr_3_:Eu GC shown in Figure 1g, which may originate from the increased electron density around Pb^2+^ after Eu^3+^ doping, since stronger bonding interaction between Eu^3+^ and Br^⁻^ may result in less electron donation from Pb^2+^ to Br^⁻^ and thereby increase electron density around Pb^2+^.^[^ ^38^ ^]^ This result indicates Eu^3+^ ions occupy the Pb^2+^ sites in the CsPbBr_3_ lattice during the CsPbBr_3_ crystallization under high temperature, since the ionic radius of Pb^2+^ and Eu^3+^ are comparable (Pb^2+^: 119 pm, Eu^3+^: 95 pm). The XPS spectrum of Eu^3+^ 3d_5/2_ shown in Figure 1h with binding energy at about 1135 eV is almost symmetric and can be fitted perfectly with one gaussian‐component suggesting only one chemical environment of Eu^3+^ existing either in glass matrix or in the Pb^2+^ lattice sites of CsPbBr_3_ QDs. Based on the above analysis, we reason that the lightly doped Eu^3+^ (1.5%) ions are almost entirely incorporated into the CsPbBr_3_ lattice. Due to the unequal charge of Pb^2+^ and Eu^3+^, the Eu^2+^ is also generated in GC as evidenced by the Eu^2+^ 3d_5/2_ spectrum with binding energy at 1125 eV (Figure 1h) in order to keep the charge neutrality.^[^ ^39^, ^40^ ^]^ This phenomenon can be explained by the charge compensation model based on substitution defect mechanism^[^ ^40^ ^]^ giving rise to reduction of Eu^3+^ to Eu^2+^. Figure 1i shows photoluminescence (PL) spectra of CsPbBr_3_:*x*Eu GC as a function of Eu concentration. The PL intensity of CsPbBr_3_ increases gradually as the increase of Eu content because of the promoted nucleation of CsPbBr_3_ QD leading to more specially‐confined dots with smaller sizes,^[^ ^41^, ^42^ ^]^ also reflected by TEM observation in Figure 1b–d delivering 65% PL yield for CsPbBr_3_:1.5%Eu GC. However, the characteristic Eu^3+^ emission (inset in Figure 1i) is almost suppressed but still observable. This is in contrast with some previous reports of Eu doped CsPbX_3_ nanocrystal or glass ceramic, in which the emission intensity of Eu is comparable with or even stronger than that of CsPbX_3_.^[^ ^32^, ^33^, ^38^, ^43^, ^44^, ^45^ ^]^ This discrepancy is likely attributed to our excellent crystallization of CsPbBr_3_ promoted by Eu dopant, which makes the band‐to‐band emission of CsPbBr_3_ extremely strong, and thereby the Eu intensity seems barely observable. The transparent matrix also serves as inherent protection for perovskite quantum dots. As shown in Video S1, Supporting Information, the PL emission of CsPbBr_3_ nanocrystal film is immediately quenched when dropped into boiling water, while our CsPbBr_3_:Eu GC is barely affected, proving its toughness under extreme conditions. Besides, the toxic lead is also kept from dissolving into water with the encapsulation of glass matrix, as confirmed by the inductively coupled plasma atomic emission spectrometry (ICP‐AES) measurement. (Table S1, Supporting Information). It showed that only 0.093 mg L^−1^ of Pb^2+^ was traced in water after the CsPbBr_3_:Eu GC was kept in water for 1 h, while 2.06 mg L^−1^ of Pb^2+^ was detected for the conventional CsPbBr_3_ nanocrystal film under the same circumstance.

To access its potential as X‐ray scintillator, we conducted the radioluminescence related experiments. **Figure** **2**a gives the RL spectra of CsPbBr_3_ GC with and without Eu dopant under X‐ray excitation. The RL intensity of CsPbBr_3_ GC at 530 nm enhances significantly with Eu^3+^ doping, consistent with the PL results. Different from the PL spectra, the relatively strong characteristic emissions of Eu^3+^ with peaks at 595, 616, 654, and 701 nm, which can be assigned to 5D_0_ → 7F_j_ (*J* = 1, 2, 3, 4)^[^ ^46^, ^47^ ^]^ transitions of Eu^3+^ ions respectively, become more distinct under X‐ray excitation. This feature brings an extra advantage that the overlap between RL spectra and absorption is largely reduced leading to decreased self‐absorption,^[^ ^48^ ^]^ while the stokes shift of mere CsPbBr_3_ perovskite is very small due to its direct bandgap nature. The integrated RL intensity of CsPbBr_3_:Eu GC as a function of incident X‐ray dose rate is shown in Figure 2b revealing a super‐linear relation, which is beneficial for obtaining good X‐ray image contrast. The discrepancy between PL and RL for CsPbBr_3_:Eu GC is possibly associated with the different excitation mechanism under UV and X‐ray. The incident X‐ray photons first interact with the heavy atoms of CsPbBr_3_ via photoelectric effect and Compton scattering. Note that the molar ratio of CsPbBr_3_ and Eu_2_O_3_ (Eu^3+^ source) is ≈17:1.5, thus CsPbBr_3_ QDs are the major contributors of X‐ray absorption as shown in Figure 2c. Once the X‐ray photons are absorbed, they will be first converted to high‐energy ejected electrons. These electrons with high kinetic energies move and scatter though their surrounding materials, loosing energy progressively by causing other secondary electrons, and then are transported to the luminescent centers (CsPbBr_3_ and Eu^3+^ ions in this case).^[^ ^1^, ^49^, ^50^ ^]^ In our case, we suppose that many of the hot and secondary electrons originated from CsPbBr_3_ are captured by the Eu^3+^ luminescent centers since the Eu^3+^ are incorporated into CsPbBr_3_ crystal lattice. This conjecture can be confirmed by comparing the RL intensity of reference glass sample with the same amount of 1.5%Eu and CsPbBr_3_:1.5%Eu GC, in which the Eu^3+^ RL intensity is more intense than the reference glass sample as shown in Figure S4, Supporting Information. Figure 2d gives the estimation of the X‐ray light yield of the perovskite glass‐ceramic scintillator under steady state X‐ray illumination, the reference sample was LuAG:Ce which had similar light decay constant to perovskite scintillators. It demonstrated the CsPbBr_3_:1.5%Eu GC scintillator had an estimated X‐ray light yield (steady‐state) of 10 100 photons per MeV. The light yield describes the X‐ray to photon conversion efficiency, and is considered as an internal quantum efficiency, an analogy to PL quantum yield. Hence its value significantly depends on how the X‐ray absorption is determined, which is sort of ambiguous here. Herein we treated the whole perovskite GC as the X‐ray absorber although the glass matrix was non‐emissive, in such a way that the value of 10 100 photons per MeV was obtained. The ultimate output light intensity depends on not only light yield but also X‐ray absorption coefficient and light outcoupling efficiency. Fast light decay of perovskite scintillator is considered as one of the major advantages over the conventional scintillators.^[^ ^1^, ^2^, ^3^, ^19^ ^]^ One might have the concern of whether this unique feature is still kept with Eu doping. The results in Figure 2e relieve us that the CsPbBr_3_:1.5%Eu GC has almost as fast light decay as non‐doped CsPbBr_3_, both clearly outperforming commercial CsI:TI. The measurement was conducted with an industrial test kit in Hamamatsu scintillator department, with which the X‐ray source can be immediately shut off by instantly shifting the electron beam off the metal target. The time correlated single photon counting measurement in Figure S5, Supporting Information, indicates the decay time of CsPbBr_3_:1.5%Eu GC and CsPbBr_3_ GC are 6.78 and 2.89 ns respectively, both in the nanosecond regime. Finally, Figure 2f shows the photographs of CsPbBr_3_:Eu GC scintillations under X‐ray excitation exhibiting strong and uniform emissions. Table S2, Supporting Information, illustrates the figures of merits of perovskite glass ceramic scintillator, together with previously reported perovskite scintillators and some typical commercial scintillators. Despite decades of intensive research of inorganic scintillators, there is not yet a single material that combines all the figures of merits, instead people have developed many scintillators for different applications. The perovskite GC scintillator, though not ideal, has balanced parameters of moderate light yield and fast light decay, which might be useful in some of the real applications, for example, dynamic X‐ray imaging or even medical CT that requires high time resolution and high stability.

**Figure 2:**
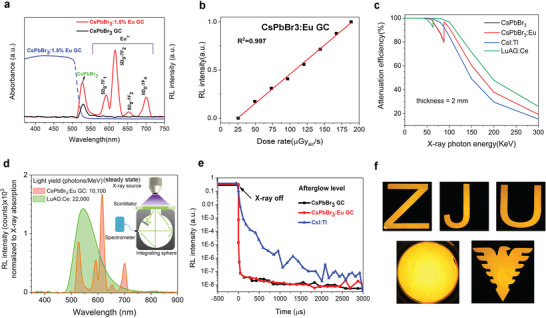
Characterizations of CsPbBr_3_:Eu GC as X‐ray scintillator. a) Radioluminescence (RL) spectra of CsPbBr_3_ GC, CsPbBr_3_:1.5%Eu GC (optimal concentration of Eu dopant) under X‐ray excitation with a dose rate of 189 µGy_air_ s^−1^ at a voltage of 50 kV. The absorption spectra is also included to demonstrate the self‐absorption issue. b) RL intensity dependence on X‐ray irradiation. c) X‐ray attenuation efficiency of CsPbBr_3_ GC, CsPbBr_3_:1.5%Eu GC, commercial CsI:TI, and LuAG:Ge. d) Steady‐state light yield calculation of our optimized CsPbBr_3_:1.5%Eu GC, commercial LuAG:Ge, inset figure is the measurement setup with integrating sphere. e) Afterglows of CsPbBr_3_ GC scintillators with and without Eu dopant, compared with widely‐used CsI:TI commercial scintillator. f) Photograph of CsPbBr_3_:1.5%Eu GC under X‐ray illumination (dose rate of 189 µGy_air_ s^−1^, voltage: 50 KV).

The above characterizations imply the potential of CsPbBr_3_:Eu GC as scintillator for X‐ray imaging. Herein, we built a home‐made X‐ray imaging system in **Figure** **3**a, and successfully acquired X‐ray images of chip A (optical photograph shown in Figure 3b) by using CsPbBr_3_ GC without and with Eu^3+^ doping. As expected, the image quality using CsPbBr_3_:Eu GC is clearly better than the one using non doped CsPbBr_3_ GC, due to the enhanced RL intensity and reduced light scattering (Figure 3c). The X‐ray image of chip B acquired by conventional CsPbBr_3_ nanocrystal film reveals quite weaker image sharpness because of the strong optical crosstalk induced by light scattering (Figure S6, Supporting Information). The modulation transfer functions (MTF)^[^ ^51^ ^]^ of images obtained from CsPbBr_3_ GC, CsPbBr_3_:Eu GC, and CsPbBr_3_ nanocrystal film have been calculated by the slanted‐edge method^[^ ^52^ ^]^ to compare the spatial resolution (Figure 3d). X‐ray edge images used for MTF calculation are shown in Figure S7, Supporting Information. The spatial resolution is defined to be the spatial frequency (lp mm^−1^) at MTF = 0.2. As a result, the image resolution with CsPbBr_3_:Eu GC scintillator is 15.0 lp mm^−1^ which is much larger than 4.1 lp mm^−1^ for CsPbBr_3_:GC, while conventional CsPbBr_3_ nanocrystal film only gives 1.5 lp mm^−1^. To further confirm those values, we took images of the standard X‐ray resolution test pattern plate (Figure 3e), showing the observation limit was between ≈14 and 16 lp mm^−1^, consistent with its calculated MTF value. The corresponding X‐ray images of standard test pattern plate for referencing CsPbBr_3_ GC and CsPbBr_3_ nanocrystal film are presented in Figure S8, Supporting Information. It is noted that the referencing CsPbBr_3_ nanocrystal film was reported with 9.8 lp mm^−1^ in the system with scintillator screen very close to the photodiode array,^[^ ^2^ ^]^ such that the optical crosstalk of scattered scintillation light makes minimum impact. In our case, the camera is placed away from the scintillator screen in order to increase field of view, and we should expect even better resolution if the transparent GC scintillator is attached closely to the CMOS sensor of same pixel size. Even so, this is still the highest spatial resolution for perovskite‐based X‐ray imaging^[^ ^1^, ^2^ ^]^ to the best of our knowledge, it is even superior to 10 lp mm^−1^ of the typical commercial CsI (TI) scintillator based X‐ray imaging.^[^ ^16^ ^]^

**Figure 3:**
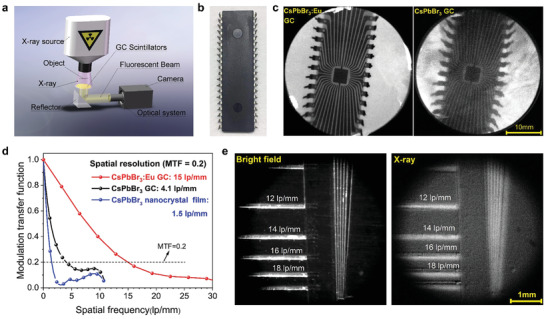
Spatial resolution evaluation of X‐ray imaging based on three scintillators: CsPbBr_3_ nanocrystal film, CsPbBr_3_ GC, and CsPbBr_3_:1.5%Eu GC. a) The schematic of X‐ray imaging system. b) Photograph of chip A. c) X‐ray images of chip A using CsPbBr_3_ GC scintillators with and without Eu^3+^ doping (dose rate: 47.2 µGy_air_ s^−1^, voltage: 50 KV). d) Modulation transfer functions (MTF) of X‐ray images obtained from CsPbBr_3_ GC scintillators with and without Eu^3+^ doping, the conventional CsPbBr_3_ nanocrystal film scintillator is set as reference. The image's spatial resolutions (when MTF value equals 0.2) acquired from CsPbBr_3_ GC, CsPbBr_3_:Eu GC, and CsPbBr_3_ QDs scintillators are 15.0, 4.1, and 1.5 lp mm^−1^, respectively. e) Bright field and X‐ray image of standard X‐ray resolution test pattern plate using CsPbBr_3_:Eu GC scintillator (dose rate: 189 µGy_air_ s^−1^, voltage: 50 KV).

The X‐ray radiation stability, thermal and environmental stability of CsPbBr_3_:Eu GC scintillator has been systematically investigated in terms of RL, X‐ray image quality. The RL intensity has retained almost 100% of its initial value following continuous X‐ray irradiation for 100 h and thermal annealing for another 100 h under 85 °C, clearly outperforming the conventional nanocrystal film shown in **Figure** **4**a. The relative humidity was also recorded every 10 h showing a humid ambient of the testing environment (RH: ≈40% and 80%). The corresponding X‐ray images of chip B was acquired at three specific timing during the aging process (0, 100, and 200 h), which gave nearly identical sharpness visually (Figure 4b). X‐ray images of chip B obtained at every 10 h during the stability test are presented in Figure S9, Supporting Information. The stability tests demonstrate robustness of our CsPbBr_3_:Eu GC as scintillator against harsh ambient environment (high humidity, thermal annealing) and longtime X‐ray illumination. Figure 4c shows high‐quality X‐ray image of human finger with obvious biological tissue phase contrast and clear joint details benefited from the low optical crosstalk of the scintillation. Figure 4d,e are the X‐ray images of different circuit boards with various electronic components. To access the suitability of CsPbBr_3_:Eu GC scintillator for the real‐time X‐ray imaging, a video‐rate X‐ray imaging of artificial bone bending (Video S2, Supporting Information) was made which exhibited a distinct phase contrast without ghost imaging effect. Moreover, we monitored the incubation process of a fertilized quail egg by acquiring its X‐ray images during the incubation process (Figure S10, Supporting Information). The image obtained at the 12th day showed obvious phase contrast and a pair of wings appeared indistinctly. These X‐ray imaging demos demonstrate great potential of CsPbBr_3_:Eu GC scintillator for the medical or industrial X‐ray imaging application.

**Figure 4:**
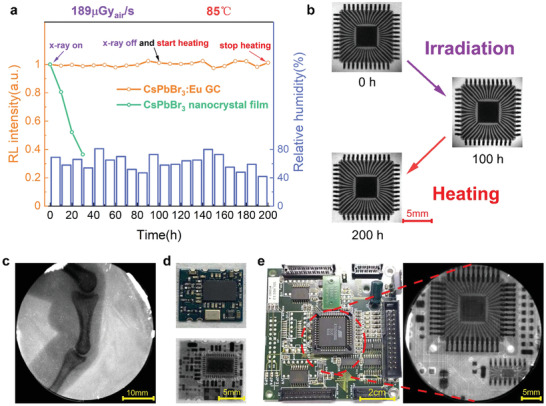
Stability test and X‐ray imaging demos of objects of interest. a) RL intensity monitoring of CsPbBr_3_:Eu GC and conventional CsPbBr_3_ nanocrystal film under X‐ray illumination and thermal stressing in humid air: X‐ray irradiation for the initial 100 h (dose rate of 189 µGy_air_ s^−1^, voltage: 50 KV), and subsequently another 100 h heating under 85 °C, the moisture level is recorded as well. The intensity is measured with integrating sphere. b) The X‐ray images of chip B acquired at three different stages (0, 100, and 200 h). c) X‐ray image of atificial finger (dose rate: 189.0 µGy_air_ s^−1^, voltage: 50 KV). d) Photograph of circuit board A (top) and its X‐ray image (below) (dose rate: 47.2 µGy_air_ s^−1^, voltage: 50 KV). e) Photograph of a circuit board B (left) and X‐ray image of its central part encircled by red dash lines (right) (dose rate: 47.2 µGy_air_ s^−1^, voltage: 50 KV).

In conclusion, we have successfully developed an Eu doped perovskite glass‐ceramic scintillator to eliminate the scintillation scattering and improve operational stability. The incorporation of Eu promotes the crystallization of CsPbBr_3_ QDs, and makes them distribute uniformly in the matrix. As a result, high resolution X‐ray imaging of 15 lp mm^−1^ is realized. In the meanwhile, the operational and environmental stability of CsPbBr_3_:Eu GC scintillators are significantly enhanced. This approach and platform could also be applicable to other perovskite or perovskite derivative scintillators that might possess better figures of merits than CsPbBr_3_ scintillators.

## Conflict of Interest

The authors declare no conflict of interest.

## Supporting information

Supporting Information

Supplemental Video 1

Supplemental Video 2

## Data Availability

Research data are not shared.
