# Supplementary material for: Highly Resolved and Robust Dynamic X‐Ray Imaging Using Perovskite Glass‐Ceramic Scintillator with Reduced Light Scattering
Source: Adv Sci (Weinh). 2021 Jun 2;8(15):2003728. doi: 10.1002/advs.202003728 (PMC8336613; doi:10.1002/advs.202003728)
Supplement: Supplementary file 1 — Supporting Information [file ADVS-8-2003728-s001.pdf]

© 2021 Wiley-VCH GmbH

ADVANCED SCIENCE
Open Access

Supporting Information

for *Adv. Sci.,* DOI: 10.1002/advs.202003728

Highly Resolved and Robust Dynamic X-ray Imaging Using Perovskite Glass-ceramic Scintillator with Reduced Light Scattering

Wenbo Ma, Tingming Jiang, Ze Yang, Hao Zhang, Yirong Su, Zeng Chen, Xinya Chen, Yaoguang Ma, Wenjuan Zhu, Xue Yu, Haiming Zhu, Jianbei Qiu, Xu Liu, Xuhui Xu, and Yang (Michael) Yang**

Supporting Information

Highly resolved and robust dynamic X-ray imaging using perovskite glass-ceramic scintillator with reduced light scattering

Wenbo Ma, Tingming Jiang, Ze Yang, Hao Zhang, Yirong Su, Zeng Chen, Xinya Chen, Yaoguang Ma, Wenjuan Zhu, Xue Yu, Haiming Zhu, Jianbei Qiu, Xu Liu, Xuhui Xu, and Yang (Michael) Yang**

Materials and Methods

Sample fabrication. $CsPbBr_3$:xEu QDs glass-ceramic (GC) samples were synthesized by a melting-quenching route and subsequent crystallization (heat-treatment). The GC matrix were designed with molar compositions of 35B_2O_3 - 35SiO_2 - 12ZnO, and the added perovskite-related components were 9Cs_2CO_3 -6$PbBr_2$ -3NaBr-(6-x)Eu_2O_3 (in mol%). The raw materials of B_2O_3 (99%), SiO_2 (99.99%), ZnO(99.9%), Cs_2CO_3(99%), $PbBr_2$(99.9%), NaBr(99.99%) and high purity Eu_2O_3 (99.99%) were well mixed and ground into powders with an agate mortar and pestle. Then the well-ground stoichiometric compounds were put into an alumina crucible and melted at 1200 °C for 20 min in air atmosphere. After that, the melt was poured onto a 420°C pre-heated stainless steel plate and then pressed by another brass plate to form precursor glass. In addition, the glasses are annealed in a muffle furnace at 420°C for 3 hours to release the thermal stress, precursor glasses (PG) were formed. Subsequently, PG were heat-treated for 17 h at 500 °C to form transparent GCs. The Eu doped $CsPbBr_3$ GC products were optical polished or ground into powders for further characterization and usage.

Material characterization. The phase samples were identified via XRD measurement (D8ADVANCE/Germany Bruker X-ray diffractometer), with Cu-Kα radiation (λ=0.15405 nm) in the 2θ range from 10° to 60°. The microstructures of the GCs were analyzed by transmission electron microscopy (TEM) and high-resolution field transmission electron microscopy (HRTEM) using U.S. FEI TecnaiG2 F20 operating at 200 kV. The photo-

luminescence (PL) spectra were measured with a HITACHI F-7000 fluorescence spectrophotometer using a 150 W Xe lamp as the excitation source. The afterglow and PL decay were detected using a home-setup microfluorescence system. The excitation light (515nm) was generated by femtosecond laser, (Light Conversion Pharos, 1030 nm, <300 fs, 1 MHz). TRPL decay kinetics were collected using a TCSPC module (PicoHarp 300) and a SPAD detector (IDQ, id100). The transmittance spectra and absorption spectra were recorded in the wavelength range from 200 to 800nm using a Model HITACHI U-4100 type spectrophotometer (Hitachi, Tokyo, Japan).

The X-ray attenuation efficiency (AE) could be calculated using the following formula:

$$AE(\%) = \left(1 - e^{-c(E)\rho d}\right) \times 100\%$$

where c(E) is the photon cross section function obtained from the XCOM database of National Institute of Standards and Technology (NIST), E is the corresponding photon energy, ρ is the density of scintillator and d is the thickness.

The commercial LuAG:Ce scintillator of light yield 22000 photons/MeV was used as a reference to calibrate the light yields of organic scintillators. Another CsI:TI commercially available scintillator of light yield of 57000 photons/MeV was used as second reference for double check. The $CsPbBr_3$:xEu QDs glass-ceramic (GC) samples and the reference scintillators were set at the same position to measure the RL spectra. Then the corresponding photon counting ($PC_{measured}$) results were obtained by integrating these RL spectra. The measurement setup is illustrated in the inset figure of fig. 2d. The X-ray absorption coefficient used for light yield calculation is at 22keV, which is the main X-ray output of our X-ray tube. Light yield is defined as the ratio of photon numbers emitted from the luminescent sites to the total absorbed X-ray energy, it represents an internal X-ray conversion efficiency. In view of this definition, the emission photon counts of scintillators should be normalized to same X-ray attenuation (100%) as the following formula:

$$PC_{normalized} = \frac{PC_{measured}}{AE\%}$$

where AE% is the X-ray attenuation efficiency (%) of scintillators with certain thickness as described in the last section. The light yield of $CsPbBr_3$:xEu QDs glass-ceramic (GC) ($LY_{perovskite}$) can be calculated by the following formula:

$$LY_{perovskite} = LY_{ref.} \cdot \frac{PC_{normalized}(Perovskite)}{PC_{normalized}(ref.)}$$

X-ray imaging. The X-ray source was Mini-X X-ray tube (target material: Ag) supplied by Amptek Inc and operated at 50 KV, exhibiting the X-ray output spectrum with both intense peak and average photon energy at about 22 KeV. The current of tube was tuned between 5 μA and 79 μA to modulate the X-ray dose rates. The objects for imaging and GC scintillators were placed perpendicular to the incident X-ray, and the scintillators were fixed just behind the objects. In order to extinguish the negative influence induced by direct radiation from X-ray source to camera, a reflector was used to deflect the optical path by 90 degrees. Lastly, a CMOS camera (Photometrics 95B) was used to collect X-ray images. This camera consists of 1200×1200 pixels and the area of each pixel is 11μm × 11 μm.

MTF measurements. MTF determines the spatial resolution of imaging system and represents the ability to transfer input signal modulation of spatial frequency relative to its output. An MTF value of 1 indicates the perfect detection of a given spatial frequency. Using slanted-edge method to calculate MTF, we took the X-ray images of a sharp edge from a piece of aluminum (thickness: ~1mm), and the X-ray dose rate was 47.2 μGyair/s. Next the edge spread function (ESF) was derived by the edge profile, from which we could deduce the line spread function (LSF) by calculating derivative. Finally, the Fourier transform of the LSF defines the MTF, meaning the MTF curves could be calculated by the following formula:

$$MTF(\nu) = \mathcal{F}(LSF(x)) = \mathcal{F}\left(\frac{dESF(x)}{dx}\right)$$

Where the ν is spatial frequency, x is the position of pixels. Due to using different optical system, the position of pixels (x) are defined by following formula:

$$x = \frac{N \cdot d}{\beta}$$

Where the N is the ordinal number of pixels in X-ray edge image, d is the pixel size (11 μm) and β is optical magnification.

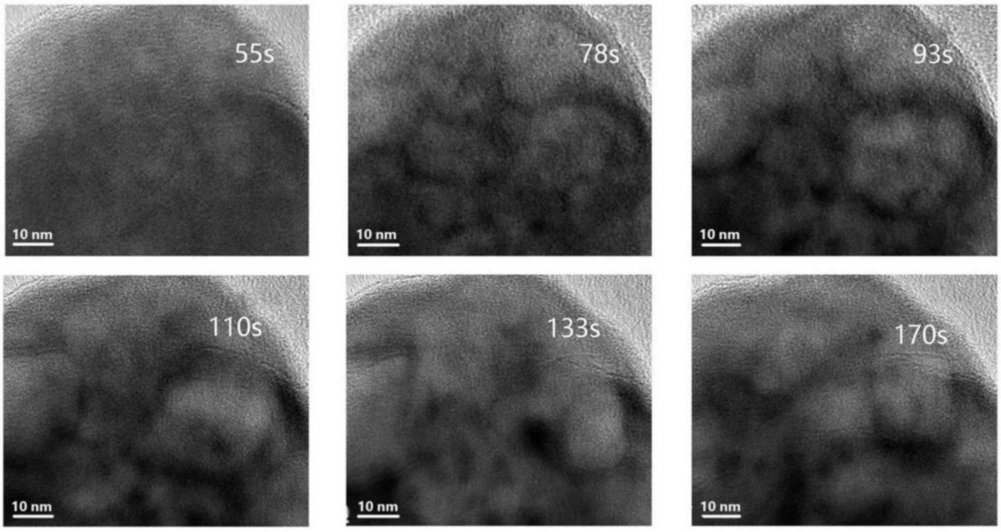

Figure S1. TEM images of in-situ growth of $CsPbBr_3$ QDs inside PG sample without Eu dopant induced by high-energy electron beam.

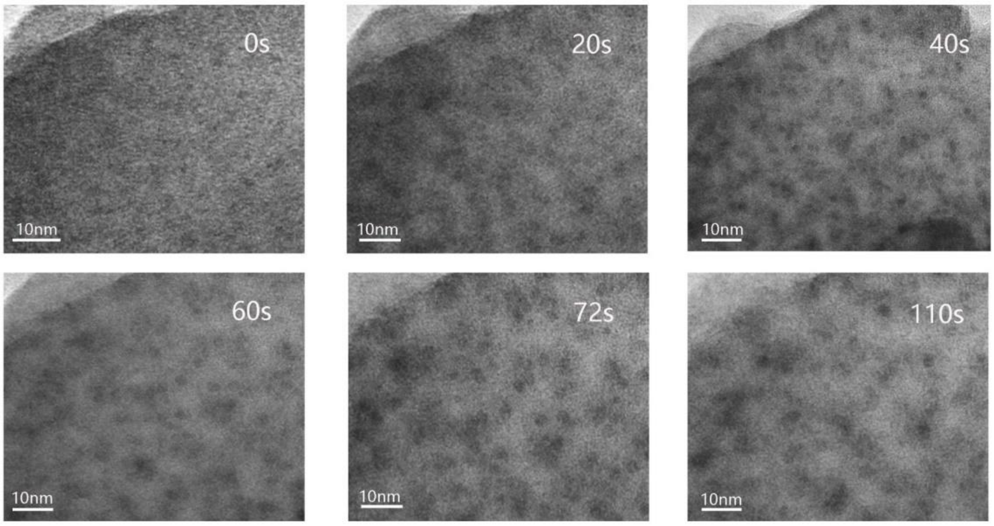

Figure S2. TEM images of in-situ growth of $CsPbBr_3$ QDs inside PG sample with Eu dopant induced by high-energy electron beam.

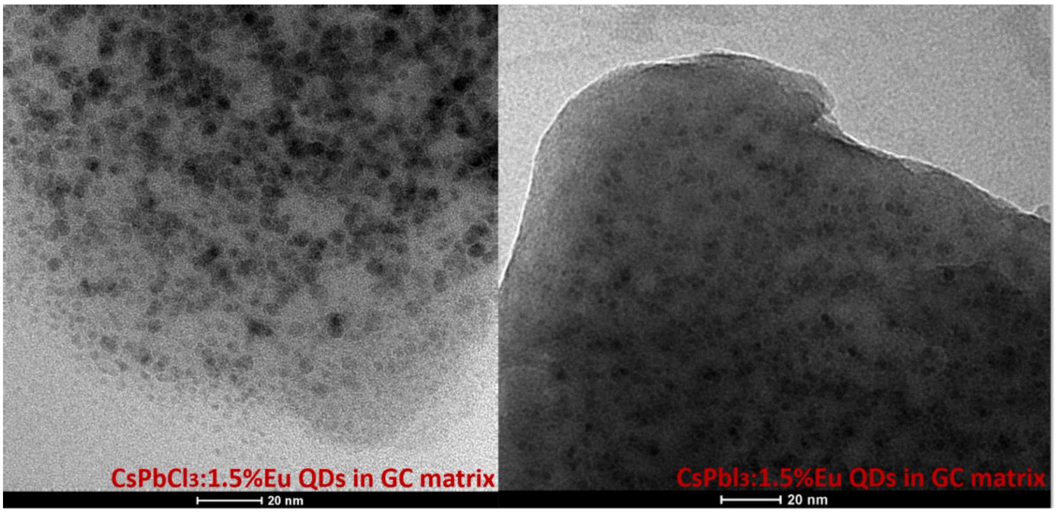

Figure S3. TEM images of $CsPbCl_3$:1.5% Eu QDs and $CsPbI_3$:1.5% Eu QDs in GC matrix.

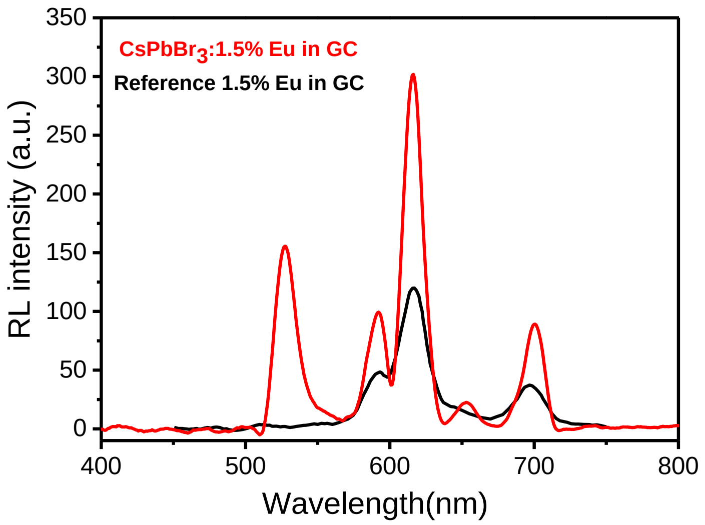

Figure S4. RL spectra of $CsPbBr_3$:1.5% Eu GC and RL spectra of only 1.5% Eu in glass, measured with integrating sphere under same input X-ray intensity.

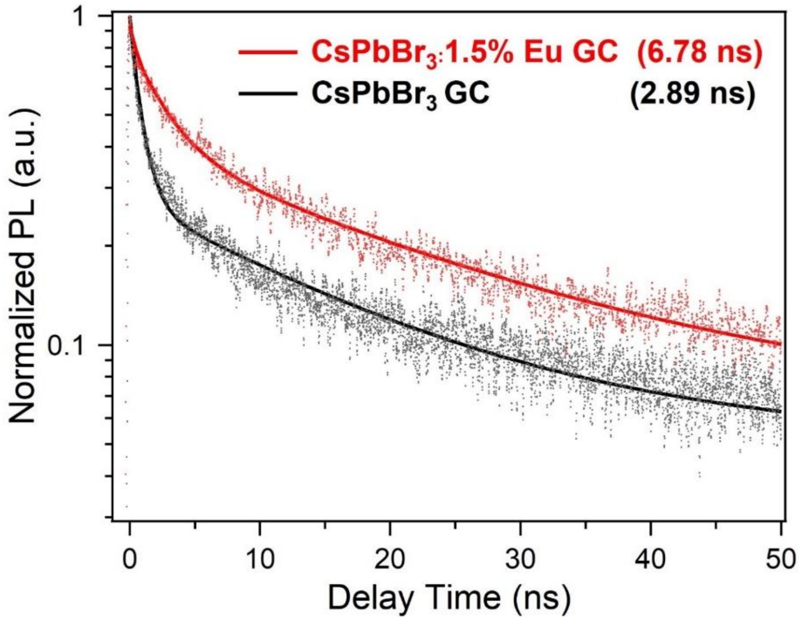

Figure S5. PL decay of $CsPbBr_3$ GC with and without Eu dopant.

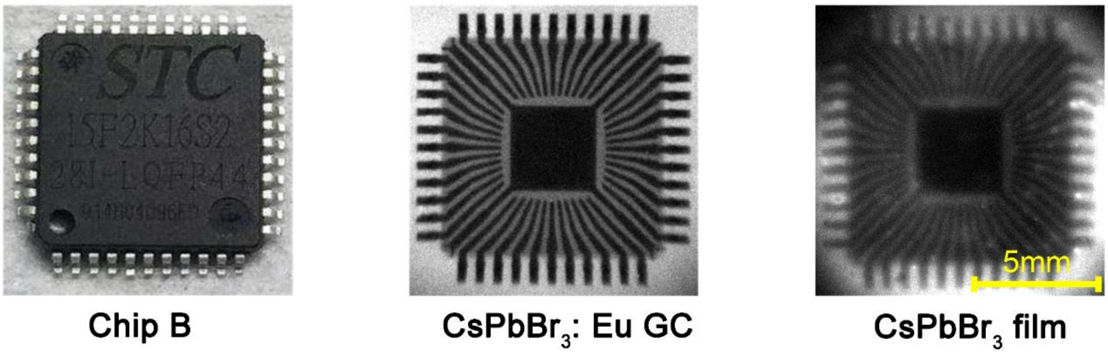

Figure S6. X-ray images of chip B acquired by $CsPbBr_3$:Eu GC scintillator and conventional $CsPbBr_3$ nanocrystal film.

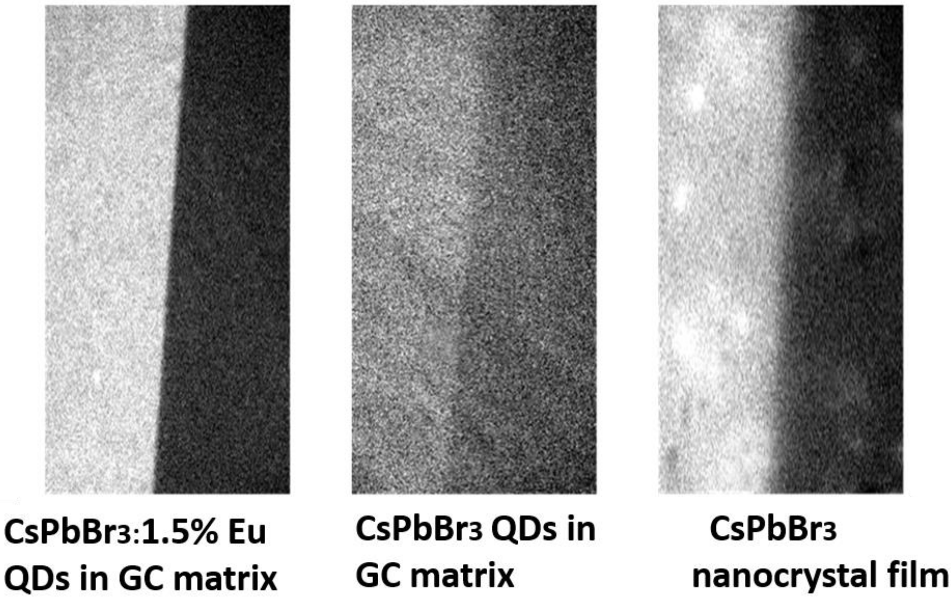

Figure S7. X-ray edge images used for MTF calculation, acquired by $CsPbBr_3$:1.5%Eu GC scintillator, $CsPbBr_3$ GC scintillator and $CsPbBr_3$ nanocrystal film scintillator, respectively.

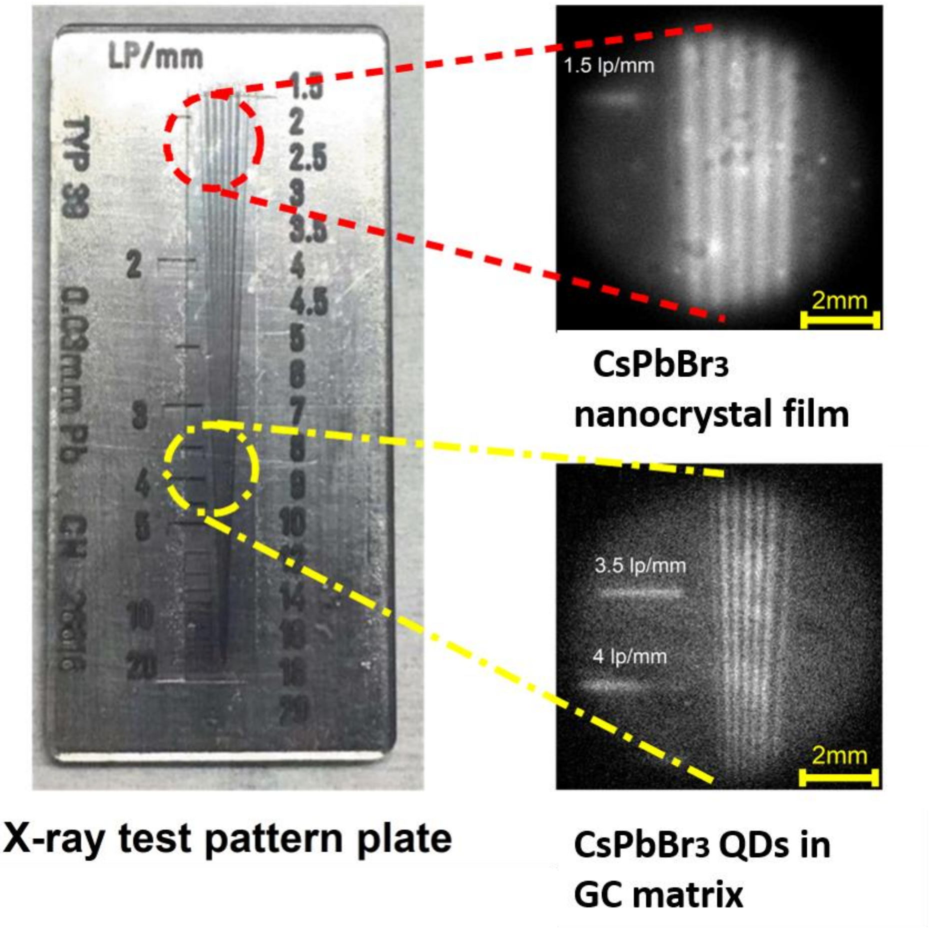

Figure S8. X-ray images of test pattern plate utilizing $CsPbBr_3$ nanocrystal film and $CsPbBr_3$ GC scintillator.

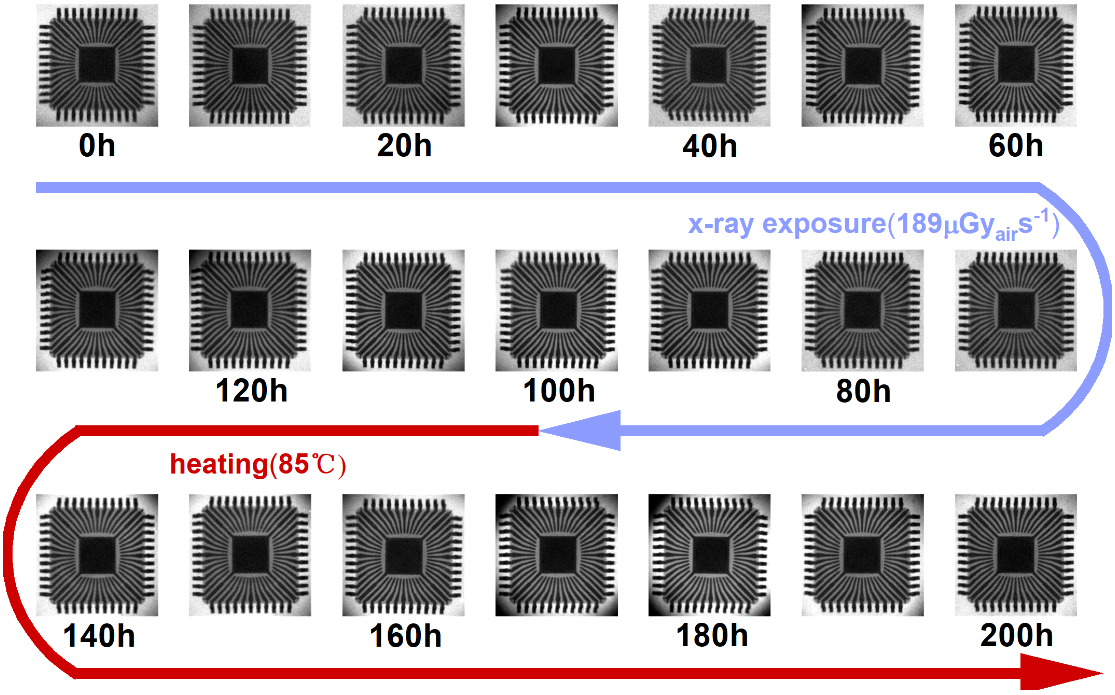

Figure S9. X-ray images of chip B acquired by $CsPbBr_3$:Eu GC every 10 h during the stability process.

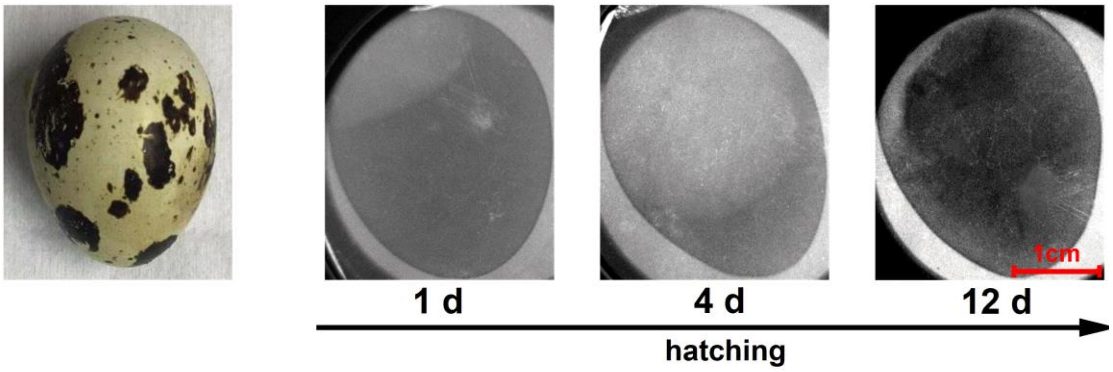

Figure S10. X-ray images of a fertilized quail egg acquired by $CsPbBr_3$:Eu GC scintillator at different time during the incubation process.

Table S1. The solubility of Pb^{2+} in water. $CsPbBr_3$:Eu GC and $CsPbBr_3$ nanocrystal film are dipped in water under room temperature for 1 hours. The Pb^{2+} solubility is measured by inductively coupled plasma atomic emission spectrometry (ICP-AES).

**Sample**	**Pb^{2+} solubility (mg/ml)**
$CsPbBr_3$:Eu GC	0.093
$CsPbBr_3$ nanocrystal film	2.06

Table S2. Performances and parameters of conventional and perovskite scintillators. In status, **P** is phosphor, **C_1** is crystal, **C_2** is ceramic and **N** is nanocrystal. All the parameters are reported under room temperature.

Scintillator	Linear absorption coefficients to 50 KeV (cm^{-1})	Light yield (photons/MeV)	Decay time (ns)	Main emission wavelength (nm)	Status	Ref
**GOS(Tb)**	**21.15**	**70000**	**6×10^5**	**545**	**P**	**1, 2, 3**
**CsI(Tl)**	**57.56**	**54000**	**1000**	**560**	**C_1**	**4, 5**
**$CdWO_4$**	**48.39**	**19700**	**2000**	**495**	**C_1**	**2, 6, 7**
**$(Y,Gd)_2O_3$:Eu,Pr**	**24.56**	**19000**	**1×10^6**	**610**	**C_1**	**1, 2**
**SrI_2:Eu**	**48.34**	**85000**	**1200**	**422**	**C_1**	**8**
**$CsPbBr_3$ (nanocrystals)**	**35.07**	**—**	**44.6**	**530**	**N**	**9**
**$CsPbBr_3$ (nanosheets)**	**35.07**	**21000**	**2-13**	**460-550**	**N**	**10**
**$CsPbBr_3$ @Cs_4PbBr_6 () (core-shell)**	**> 35.07**	**6000**	**3**	**530**	**N**	**11**
**Rb_2CuBr_3**	**16.38**	**91056**	**4.11×10^4**	**385**	**C_1**	**12**
**$CsPbBr_3$:1.5%Eu (glass ceramic)**	**35.81**	**40100**	**6.78**	**530, 613**	**N/C_2**	**This work**

Reference:

1. Moses WW. Scintillator requirements for medical imaging. (1999).

2. Deych R, Dolazza E. New trends in X-ray CT imaging. In: *Radiation detectors for medical applications*. Springer (2006).

3. Tian Y, Cao W-H, Luo X-X, Fu Y. Preparation and luminescence property of Gd_2O_2S: Tb X-ray nano-phosphors using the complex precipitation method. *Journal of Alloys and Compounds* **433**, 313-317 (2007).

4. Sakai E. Recent measurements on scintillator-photodetector systems. *IEEE Transactions on Nuclear Science* **34**, 418-422 (1987).

5. Schotanus P, Kamermans R. Scintillation characteristics of pure and Tl-doped CsI crystals. *IEEE Transactions on Nuclear Science* **37**, 177-182 (1990).

6. Kinloch D, Novak W, Raby P, Toepke I. New developments in cadmium tungstate. *IEEE transactions on nuclear science* **41**, 752-754 (1994).

7. Maddalena F, *et al.* Inorganic, organic, and perovskite halides with nanotechnology for high–light yield x-and γ-ray scintillators. *Crystals* **9**, 88 (2019).

8. Cherepy NJ, *et al.* Strontium and barium iodide high light yield scintillators. *Applied Physics Letters* **92**, 083508 (2008).

9. Chen Q, *et al.* All-inorganic perovskite nanocrystal scintillators. *Nature* **561**, 88-93 (2018).

10. Zhang Y, *et al.* Metal Halide Perovskite Nanosheet for X-ray High-Resolution Scintillation Imaging Screens. *ACS Nano* **13**, 2520-2525 (2019).

11. Cao F, *et al.* Shining Emitter in a Stable Host: Design of Halide Perovskite Scintillators for X-ray Imaging from Commercial Concept. *ACS Nano*, (2019).

12. Yang B, *et al.* Lead- Free Halide Rb_2CuBr_3 as Sensitive X- Ray Scintillator. *Advanced Materials* **31**, 1904711 (2019).
