# Supplementary figures and images for: Highly Resolved and Robust Dynamic X‐Ray Imaging Using Perovskite Glass‐Ceramic Scintillator with Reduced Light Scattering
Source: Adv Sci (Weinh). 2021 Jun 2;8(15):2003728. doi: 10.1002/advs.202003728 (PMC8336613; doi:10.1002/advs.202003728)

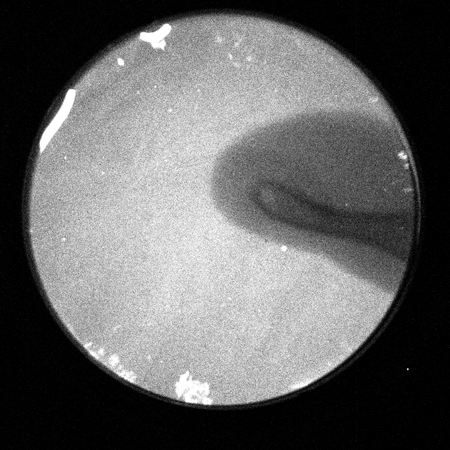

Supplement: Supplementary file 3 — Supplemental Video 2 [file ADVS-8-2003728-s002.gif]
